# Measuring patient acuity and nursing care needs in South Korea: application of a new patient classification system

**DOI:** 10.1186/s12912-022-01109-4

**Published:** 2022-11-29

**Authors:** Jeounghee Kim, TaeRim Kang, Hyun-Ju Seo, So-Young Seo, Myoungsook Kim, Youngsun Jung, Jinhyun Kim, Jung- Bok Lee

**Affiliations:** 1 Department of Nursing, Asan Medical Center, Seoul, Republic of Korea; 2 College of Nursing, Chungnam National University, 266 Munhwa-ro, Jung-gu, 301-747 Daejeon, Republic of Korea; 3 College of Nursing, Seoul National University, Seoul, Republic of Korea; 4 Department of Clinical Epidemiology and Biostatistics, Asan Medical Center, University of Ulsan College of Medicine, Seoul, Republic of Korea

**Keywords:** Patient classification system, Nursing workload, Patient acuity, Electronic health records, Nursing care needs

## Abstract

**Background:**

An accurate and reliable patient classification system (PCS) can help inform decisions regarding adequate assignments for nurse staffing. This study aimed to evaluate the criterion validity of the Asan Patient Classification System (APCS), a new tertiary hospital-specific PCS, by comparing its rating and total scores with those of KPCS-1 and KPCS-GW for measuring patient activity and nursing needs.

**Methods:**

We performed a retrospective analysis of the medical records of 50,314 inpatients admitted to the general wards of a tertiary teaching hospital in Seoul, South Korea in March, June, September, and December 2019. Spearman’s correlation and Kappa statistics according to quartiles were calculated to examine the criterion validity of the APCS compared with the KPCS-1 and KPCS-GW.

**Results:**

The average patient classification score was 28.3 points for APCS, 25.7 points for KPCS-1, and 21.6 points for KPCS-GW. The kappa value between APCS and KPCS-1 was 0.91 (95% CI:0.9072, 0.9119) and that between APCS and KPCS-GW was 0.88 (95% CI:0.8757, 0.8810). Additionally, Spearman's correlation coefficients among APCS, KPCS-1, and KPCS-GW showed a very strong correlation. However, 10.8% of the participants’ results were inconsistent, and KPCS-1 tended to classify patients into groups with lower nursing needs compared to APCS.

**Conclusion:**

This study showed that electronic health record-generated APCS can provide useful information on patients’ severity and nursing activities to measure workload estimation. Additional research is needed to develop and implement a real-world EHR-based PCS system to accommodate for direct and indirect nursing care while considering diverse population and dynamic healthcare system.

**Supplementary Information:**

The online version contains supplementary material available at 10.1186/s12912-022-01109-4.

## Background

To maintain the quality of nursing services, the nursing needs and acuity of patients should be considered when determining the level of nursing staffing [1]. An accurate and reliable patient classification system (PCS) provides a process for quantifying the care, treatment, and services required in a hospital [2]. In addition, PCSs can help make decisions about adequate assignments for nursing staff and adjust staffing to reduce costs, improve quality of care, reduce turnover rate, and improve outcomes in inpatients [3]. Although various types of PCSs have been developed, there is no single gold-standard tool that can be universally applied because of differences in healthcare and reimbursement systems.

The Korean Patient Classification System-1 (KPCS-1) was developed in 2010 and revised in 2018 for specific use in general wards, intensive care units, and neonatal intensive care units to determine appropriate nurse staffing [4]. Owing to the changing healthcare environment and aging population, the need for nursing care is increasing in acute care settings [5]. A national initiative of hospital accreditation and quality improvement of healthcare services was conducted to increase efficiency and quality, which showed that the average length of hospital stay has decreased in acute care settings [6, 7]. These factors could increase the required level of adequate nursing staff to ensure the quality of nursing care without changing the number of beds and inpatients.

However, only a few studies have compared the original and revised versions of KPCS-1, along with other tertiary hospital-specific PCSs that can help nursing administrators determine the optimal number of nursing staff required to maintain the quality of care in a given hospital. Therefore, the purpose of this retrospective study using electronic medical records was to explore the criterion validity of the Asan Patient Classification System (APCS), a new tertiary hospital-specific PCS, by comparing its rating and total scores with those of KPCS-1 and KPCS-GW, a revised version of KPCS-1.

### Nursing care in South Korea

To lay the groundwork for securing the minimum number of nursing personnel to provide an appropriate level of nursing service, the Ministry of Health and Welfare in South Korea introduced and implemented a differential nursing management fee payment system in November 1999 for the patient-to-nurse placement ratio in general nursing units [8]. However, if the standard is converted to the number of patients per shift, which does not consider the number of patients cared for by nurses working in shifts, the number of patients that one nurse must take care of during the shift increases by fivefold or more [9].

It is unrealistic to regard any outcome as optimal considering that nursing service evaluation criteria such as the differential payment system for nursing care only cover the minimal nurse/patient ratio [8, 9]. Therefore, as an alternative to the minimum staffing standards based on the number of patients, such as the simple patient-to-nurse ratio, an approach to determining staffing requirements that considers the variations of individual patients according to the need for nursing or other factors that induce workload should be developed [10]. Among the main approaches for determining the needs of individual patients, PCS is used for grouping patients according to their nursing needs and assigning the required staffing level to each group based on diagnosis or levels of acuity and/or dependency. Because only KPCS-1 has been applied, the classification system should be investigated to determine whether it can calculate the nursing workforce by reflecting real-world practice [11].

### Korean Patient Classification System

The Korean Patient Classification System (KPCS) was developed in 2009 to measure nursing workload based on the nursing needs of inpatients [12]. It was then revised in 2010 to the Korean-type Patient Classification Tool-1 (KPCS-1) after verification of reliability and validity and is the most widely used system in clinical practice. Patient classification is the process of calculating the nursing requirement score for each inpatient and classifying patients accordingly, and the patient classification score refers to the sum of each added score according to the evaluation guidelines for each item of the patient classification tool. The KPCS-1 classifies patients by focusing on a direct nursing service consisting of 12 different nursing areas (measuring vital sign, monitoring, respiratory treatment, hygiene, diet, excretion, movement, examination, medication, treatment, special treatment, and education/emotional support), 50 nursing activities, and 73 items. To increase ease of use, the number of nursing activities of KPCS-1 was reduced to 11 areas, 34 nursing activities, and 57 items. KPCS-1 was revised to KPCS-GW (General Ward) [4]. According to the total score applied to the assigned score reflecting the workload weight of each nursing activity (Additional file 1), the patients were classified into four groups: Group 1 (mild; 1–10 points), Group 2 (moderate; 11–20 points), Group 3 (severe; 21–30 points), and Group 4 (critically ill; ≥ 31 points) [4].

The National Health Insurance Corporation, one of the health authorities in Korea, modified and utilized the KPCS-1 to investigate patients’ severity/need for nursing care when determining the appropriate level of nursing workforce, including registered nurses, nurse assistants, and formal care workers, for inpatients in integrated nursing care units [13]. Therefore, the KPCS-1 might be regarded as a reference standard in the Korean context. Thus, we selected KPCS-1 and KPCS-GW for comparison with APCS.

### Asan Patient Classification System (APCS)

As a tertiary referral hospital, Asan Medical Center (Seoul, South Korea) aims to provide high-quality medical services for the efficient management of the nursing workforce while improving the calculation of nursing workload for cost-effective and appropriate nursing workforce management. Our research institute is the largest tertiary hospital in Korea with 2,700 beds and accounts for a high proportion of severe and emergent diseases such as stroke, acute myocardial infarction, and multiple major traumas. Therefore, since the KPCS and KPCS-1 do not sufficiently measure the amount of nursing work for nursing care for patients with severe diseases, we developed the APCS to measure the amount of nursing work by including a more precise scope of nursing work for direct nursing items for severely ill patients.

Accordingly, the Asan Medical Center developed the Asan Patient Classification System (APCS), which reflects the different nursing needs in each unit, and was designed and launched from 2010 to 2012. The APCS was developed by reviewing the workload management system for critical care nurses [14] and the KPCS tool [12] and establishing a consensus with the nursing management expert group and department for multiple items that are time-consuming for direct nursing. The APCS consists of 125 items across 14 areas and 101 nursing activities (Additional file 1). The development of APCS was completed by investigating whether computerized extraction is possible in targeting inpatients in internal and surgical procedures, excluding special departments such as psychiatry, emergency rooms, delivery rooms, neonatal rooms, and dialysis rooms [15]. Various types of nursing care to calculate nursing workload include direct nursing activities such as assessments, admissions and discharges, medications, medication information, respiratory, wounds, suctions, and drains. Additional activities such as nursing record documentation, identification of orders, diets, and patient/caregivers’ health education were entered into the electronic health record (EHR) of our hospital.

As for the range of patient classification scores, the range of total scores was 167–191 for APCS, 104–125 for KPCS-1, and 75–96 for KPCS-GW. The APCS reflects direct nursing activities performed in clinical practice. Further, in order to reflect the nursing needs of hospitalized patients with severe conditions, the range of patient classification scores was set to be higher than those of KPCS-1 and KPCS-GW. The nurses at Asan Medical Center regarded that dependency-acuity was increasing and that the staff number and skill mix should therefore reflect the increasing workload.

## Methods

### Study design and setting

This study retrospectively analyzed the medical records of inpatients admitted to the general ward of a tertiary teaching hospital in Seoul, South Korea.

### Participants and sampling

The study population comprised 50,314 patients admitted to 56 general medical-surgical units in March, June, September, and December 2019. Because surgery rates and hospitalizations were increased during the summer and winter seasons in Korea, PCS scores are likely to increase owing to the increase in nursing activities such as respiratory therapy and monitoring in the winter [16]. Therefore, we selected participants by quarter to exclude seasonal variations. Those who were treated in integrated care wards, intensive care units, and emergency rooms were excluded. Because clinical practice environment of them were apparently different from general wards [14].

### Data collection

Data on inpatient characteristics including department, ward, admission and discharge date, age, sex, and 125 nursing activities were collected from the electronic medical and nursing records within an existing EHR system. Patient-level patient acuity and nursing care need scores were automatically generated using the APCS, KPCS-1, and KPCS-GW by the hospital’s EHR.

### Data analysis

The collected data were analyzed using IBM SPSS Statistics for Windows, version 21 (IBM Corp., Armonk, NY, USA). Spearman’s correlation and Kappa coefficients according to quartiles were calculated to determine the criterion validity of the APCS compared with the KPCS-1 and KPCS-GW. A Spearman coefficient was interpreted as having no association (*ρ* = 0) with a perfect monotonic relationship (*ρ* =  − 1 or + 1) [17]. Strength of kappa agreement was interpreted according to Landis and Koch's as follows:0–0.20 = slight, 0.21 0.40 = fair, 0.41 0.60 = moderate, 0.61 0.80 = substantial, and 0.81–1.00 = near-perfect agreement [18].

A histogram was generated to provide a visualization of the range and comparison of EHR-generated work intensity scores associated with KPCS-1 and KPCS-GW for age groups and departments. For the general characteristics of the subjects, the frequency of each item of the APCS, KPCS-1, and KPCS-GW, and the characteristics of the patient classification score (i.e., frequency, percentage, mean, and standard deviation) were calculated. A one-way analysis of variance (ANOVA) was applied to analyze the difference in the total score of the patient classification by age group, department, and season.

### Ethics

The study was approved by the Institutional Review Board of Asan Medical Center (approval #2021–0631). The need for informed consent was waived by the Institutional Review Board of Asan Medical Center, because of the retrospective nature of the study. Patient participation was anonymous, as names or identifying information were not collected, and the study was conducted in accordance with the Declaration of Helsinki.

## Results

### Demographic data

A total of 50,314 study patients were included in the analysis, of whom 25,853 (51.4%) were male (mean age, 54.0 years; median age, 58 years). The mean length of hospital stay was 18.3 days, and the median length of hospital stay was 5 days. The average patient classification score using each PCS system was 28.3 points for APCS, 25.7 points for KPCS-1, and 21.6 points for KPCS-GW (Table 1).

**Table 1:** Characteristics of the study population (*N* = 50,314)

**Variables**	Median (interquartile range) or n (%)
**Age, years**	58 (44–68)
< 10	2,569 (5.1)
10**–**19	2,569 (5.1)
20**–**29	2,040 (4.1)
30**–**39	4,361 (8.7)
40**–**49	6,030 (12.0)
50**–**59	10,435 (20.7)
60**–**69	12,626 (25.1)
> 70	10,884 (21.6)
**Male sex**	25,853 (51.4)
**Hospital stay, days**	5 (3–11)
< 3	16,958 (33.7)
4–7	15,063 (29.9)
8–14	9,301 (18.5)
15–30	5,201 (10.3)
31–90	2,938 (5.8)
> 91	853 (5.8)
**Evaluation timing**	
March	10,929 (21.7)
June	13,032 (25.9)
September	12,635 (25.1)
December	13,718 (27.3)
**Mean PCS scores**	
APCS	28.3 ± 15.6
KPCS-1	25.7 ± 12.0
KPCS-GW	21.6 ± 10.9

### Patient classification score between APCS, KPCS-1, and KPCS-GW by age and department

**Fig. 1:**
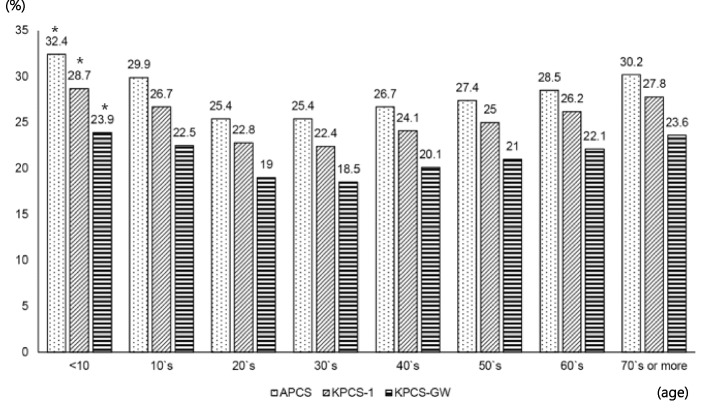
The average patient classification scores between APCS, KPCS-1, and KPCS-GW by age

When divided according to age groups, patients under 10 years of age had the highest average patient classification scores (APCS, 32.4 points; KPCS-1, 28.7 points; KPCS-GW, 23.9), while those aged 30–39 had the lowest scores (APCS, 25.4 points; KPCS-1, 22.4 points; KPCS-GW, 18.5 points). Patient classification scores were significantly different among the age groups (*p* < 0.001). In particular, the patient classification scores in the age group of 10 years and younger were significantly higher than those in the other age groups (*p* < 0.001; Fig. 1).

**Fig. 2:**
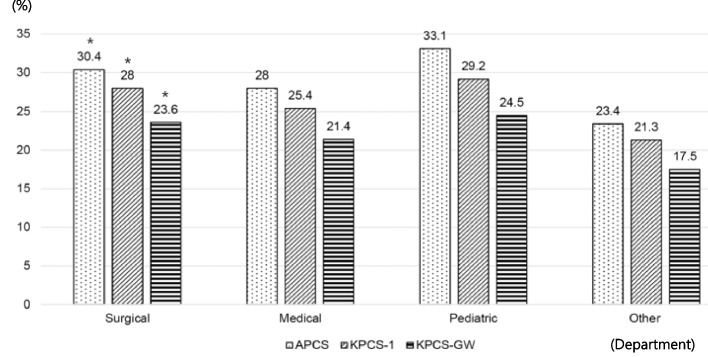
The average patient classification scores between APCS, KPCS-1, and KPCS-GW by department

When divided according to the department (i.e., medical vs. surgical vs. pediatric vs. other), those admitted to pediatric departments had the highest patient classification scores (APCS, 33.1 points; KPCS-1, 29.2 points; KPCS-GW, 24.5 points), while those admitted to “Other” departments (i.e., Otorhinolaryngology, Family Medicine, International Healthcare Center, Anesthesiology and Pain Medicine, Urology, Obstetrics and Gynecology, Ophthalmology, Psychiatry, Dentistry, Dermatology) had the lowest scores (APCS, 23.4 points; KPCS-1, 21.3 points; KPCS-GW, 17.5 points). There was a statistically significant difference in the patient classification scores of the surgical departments (*p* < 0.001); Fig. 2).

### Correlation between the APCS, KPCS-1, and KPCS-GW

The Spearman’s correlation coefficient for APCS, KPCS-1, and KPCS-GW were very strongly correlated (Spearman’s rho for APCS vs. KPCS-1; 0.98 (*p* = 0.001) and for APCS vs. KPCS-GW, Spearman’s rho 0.97 (*p* = 0.001) (Table 2).

**Table 2:** Spearman’s correlation coefficient of KPCS-1, KPCS-GW and APCS (*N* = 50,314)

	**APCS**	**KPCS-1**	**KPCS-GW**
**APCS**	1	0.98 (< .001)	0.97 (< .001)
**KPCS-1**	0.98 (< .001)	1	0.99 (< .001)
**KPCS-GW**	0.97 (.001)	0.99 (< .001)	1

### Concordance between APCS, KPCS-1, and KPCS-GW by quartiles

The kappa value (Kc) between APCS and KPCS-1 was as high as 0.91, but 10.8% of the total patient values did not match, and KPCS-1 tended to classify patients into groups with lower nursing needs compared with APCS. For example, 1211 patients were classified in the third quartile (75%) according to APCS and in the second quartile (50%) according to KPCS-1, and 101 patients were classified in the fourth quartile (100%) according to APCS and in the second quartile according to KPCS-1 (Table 3). The kappa values (Kc) of the APCS and KPCS-GW were 0.88, and 14.8% of the kappa values did not match (Table 3).

**Table 3:** Agreement between KPCS-1, KPCS-GW and APCS (*N* = 50,314)

							**APCS quartile**					
		**25%**			**50%**		**75%**		**100%**		**Total**	
		N	%		N	%	N	%	N	%	N	%
**KPCS-1 quartile**	**25%**	12,574	25.0		413	0.8	91	0.2	2	0.0	13,080	26.0
	**50%**	1,023	2.0		10,683	21.2	1,211	2.4	105	0.2	13,022	25.9
	**75%**	0	0.0		816	1.6	10,502	20.9	1,273	2.5	12,591	25.0
	**100%**	0	0.0		0	0.0	571	1.1	11,050	22.0	11,621	23.1
	**Total**	13,597	27.0		11,912	23.7	12,375	24.6	12,430	24.7	50,314	100
**KPCS-GW quartile**	**25%**	12,659	25.2		1,046	2.1	161	0.3	4	0.0	13,870	27.6
	**50%**	938	1.9		9,546	19.0	1,403	2.8	119	0.2	12,006	23.9
	**75%**	0	0.0		1,320	2.6	9,689	19.3	1,343	2.7	12,352	24.6
	**100%**	0	0.0		0	0.0	1,122	2.2	10,964	21.8	12,086	24.0
	**Total**	13,597	27.0		11,912	23.7	12,375	24.6	12,430	24.7	50,314	100

^1)^ APCS and KPCS-1 Weighted Kappa: 0.91 (95% CI: 0.907, 0.912)

^2)^ APCS and KPCS-GW Weighted Kappa: 0.88 (95% CI: 0.876, 0.881)

## Discussion

In this study, we examined the criterion validity of the APCS, a new patient classification system to estimate nursing workload using the EHR system since 2010 at a tertiary teaching hospital, compared to the KPCS-1 and KPCS-GW. Among the 50,314 patients admitted to the general ward of our hospital, the average patient classification scores were 28.3 points for APCS, 25.7 points for KPCS-1, and 21.6 points for KPCS-GW. The kappa value between APCS and KPCS-1 was 0.91, and that between APCS and KPCS-GW was 0.88. While both kappa values were quite high, the nursing needs of some patients were underestimated by the existing tools compared with APCS.

The classification of caring needs and intensity of inpatient nursing is a challenging task for nursing managers and healthcare organizations [19]. The existing prototype PCS in South Korea might not be optimal for efficiently measuring the direct nursing care activities provided to inpatients with moderate-to-severe conditions. In addition, nursing intensity measurements used in other countries are difficult to apply in the context of the Korean health system, which employs a different health reimbursement system [20, 21], patient dependency weighting [22], and the nurse-patient ratio and assignment [23, 24]. The findings of the present study indicate that capturing direct nursing care activities based on disease severity is a reliable and feasible measure compared to the existing KPCS-1 and KPCS-GW PCS.

### Comparison of APCS, KPCS-1, and KPCS-GW

The proposed APCS can be rated by data collected using EHR, whereas previous PCS tools were manually rated by staff nurses, unit managers, attending nurses, and clinical nurse experts [12, 25, 26]. The only studies applying RAFAELA PCS gathered data using medical records to classify the dependency/acuity of inpatients in general wards [27–29]. Therefore, the new APCS has the advantage of being less labor-intensive than previous tools in terms of data collection. Therefore, the implementation of APCS is feasible for identifying and estimating the current workload of nursing workforce nurses regarding nursing care needs by efficiently classifying the dependency of inpatients in real time.

Spearman's correlation analysis with APCS, KPCS-1, and KPCS-GW showed that all three instruments had a very high correlation. The results may be explained by the fact that the APCS included some nursing activities and domains derived from the KPCS-1. Regarding agreement between APCS, KPCS-1 and KPCS-GW, the kappa value between the APCS and KPCS-1 was 0.91 and the kappa value (Kc) of the KPCS-GW was 0.88. Although there was a high level of agreement, the existing tools tended to underestimate the nursing needs of patients with high acuity compared with the APCS.

The APCS, which is based on 73 KPCS nursing activities, reflects the various tasks involved in 125 nursing activities by considering the opinions of the departments that can extract nursing tasks that are actually performed through a computational process rather than manual data entry. Notably, when analyzed according to age group, the group under 10 years of age showed the highest score among age groups. As pediatric patients are characterized by their developmental stage, their age and weight are considered in various medical devices as well as drug dosages [30]. In consideration of the specificity and complexity of pediatric patients, the APCS added pediatric nursing items including spoon feeding, infant/neonate bottle feed, preparation for pediatric sleep examination procedure, infant phototherapy/infant circumcision, first aid for febrile seizures in children, breastfeeding management, and newborns. Nursing, bed bathing, and treatment for newborns were also considered.

According to Song et al. (2018) and KPCS-1, the Pediatric Patient Classification System (PPS) was developed as a pediatric patient classification tool that considers the nursing characteristics of pediatric patients [30]. PPS has been shown to be a comprehensive tool that reflects the dependency and nursing care needs of pediatric and adult patients admitted to general wards. Importantly, a study on PPS showed that the patient classification score was significantly higher in the patient group aged less than 6 years [30], which is consistent with our current results.

In this study, the age group with the second highest patient classification score after the age group under 10 years of age was the 70 or older group. For the elderly, various types of nursing activities (e.g., assisting, changing positions, and helping patients stand upright) inevitably increase owing to the presence of chronic diseases, increase in the number of medications, blood sugar tests and intravenous administrations, use of complex catheters, tube feeding, and excretion. As the importance of intensive nursing care for preventing falls and risky behaviors in elderly patients continues to grow, nursing management fees have been expanded and revised from “applying and managing physical restraint” to “risk behavior management” [31]. The presence of comorbidities, decreased ADL, cognitive impairment, and depression in elderly inpatients is an important predictor of increased nursing care [32]. Elderly risk screening and assessment tools suggest that high-risk older adults have longer hospital stays, require more nursing care, and have an increased risk of falls [33]. Lee et al. (2014) explained the burden of nursing care for elderly patients in relation to complex health issues, lack of understanding, and the possibility of falls [34]. However, as there are insufficient data on the workload of nursing for elderly patients, research on developing a patient classification system is required to estimate the need for nursing care for elderly patients and the amount of nursing work. Accordingly, it is necessary to consider the establishment of a management fee for elderly patients and the arrangement of nursing staff in the ward.

A patient's nursing need/severity is related to the amount of nursing work, and the presence of an appropriate number of nurses affects the incidence of adverse reactions, including infection, pressure sores, falls, and prescription errors [35]. An optimal nurse-to-patient ratio is important to improve the quality of care and patient outcomes [36]. In Korea, the Ministry of Health and Welfare's 2019 medical delivery system improvement policy contains content such as improvement of the evaluation and compensation system for intensive care of critically ill patients in tertiary hospitals [37]. As the number of critically ill patients receiving care in tertiary hospitals increases, the need for nursing care and nursing workload will inevitably increase. In our study, 63.2% of patients were placed in groups 3 (severe) and 4 (critically ill) according to the KPCS-1, showing that our study group had a higher proportion of patients with more severe conditions compared to those in previous work. For example, the study of Song et al. [4] reported that 30% of the patients were classified into groups 3 and 4. Therefore, the APCS might be more suitable than the existing KPCS-1 and KPCS-GW because it reflects the real-world situation of tertiary hospitals in charge of severe patients. In other words, a comprehensive APCS to capture various nursing care services and workloads for severely ill patients could be applicable to upper-level general hospitals as well as general hospitals. APCS is feasible to collect data from the EHR compared to the manual methods of other tools, despite complexity of nursing activities.

## Strengths and limitations

The APCS was developed to reflect a diverse range of nursing activities, and it was possible to calculate the nursing workload quickly and efficiently because the necessary data could be extracted from a computerized source. In addition, agreement between the new APCS and the existing Korean PCSs was near perfect, indicating high criterion validity.

However, it is possible that some nursing records or contents of nursing activities in the APCS have been omitted, because of the inadequacy of documentation related to time shortage by the nurses. In addition, although patient education and emotional support of patients often occur in a short time (< 3 min), these functions are spread throughout the day in real-life settings, patient classification tools only count activities that are more than 15 min long and only count one activity per day, which would hinder an accurate assessment of the number of educational and emotional support nursing activities provided by nurses [30]. This limitation means that it is impossible to check the qualitative data on nursing as well as the quantitative data on nursing work. Therefore, the convenience and accuracy of entering nursing records and prescriptions have improved through the application of an information record system that incorporates new technologies such as voice recognition. Through the use of ICT, the completeness of nursing records and prescriptions for nursing activities can be improved [38, 39].

Although the APCS intended to estimate the amount of nursing work by classifying the patient's severity based on the patient's need for nursing care, indirect nursing activities such as nursing student education, documentation, and handover were not sufficiently considered. Hence, the level of appropriate nursing manpower placement may be underestimated in the APCS. In addition, some nursing activities related to patient safety, such as management of novel infectious diseases (e.g., COVID-19), nursing of isolated patients, and prevention of bedsores and falls, were not included in the APCS [31]. In a study by Rauhala and Fagerstrom (2007), direct nursing work accounted for 45% of all responsibilities and non-patient care work accounted for 11% of responsibilities. It is recommended that an appropriate level of nursing staff be prioritized in consideration of the various factors affecting the health of patients [29].

## Implications for future research

In clinical practice, as the number of direct and indirect nursing tasks is increasing, the nursing situation is rapidly changing, and there is an urgent need for a supplemented nursing workload calculation method that accurately reflects nursing needs and appropriately allocates nursing personnel. To do this, the average time per nursing action should be calculated by measuring the actual nursing work, and the exact amount of nursing work and required nursing manpower should be calculated by considering the nursing record time and indirect nursing time, such as administrative work, and reflecting the legal working hours and break times. Further research and improvements are required to address this issue.

Nursing care intensity varies according to the specific needs and abilities of the patients within the patient classification system. In other words, there are growing calls to reflect changes in patients’ psychosocial status, medical status, and treatment transition, and a nursing intensity that varies according to the needs of nursing education, nursing intervention, and psychosocial needs in addition to medical diagnosis should also be developed [36, 40]. Ko and Park (2020) suggested that the optimal nursing workforce level placement is to use both (1) patient classification, which classifies patients according to their nursing needs and focuses on direct nursing work, and (2) nursing intensity, a concept of nursing workload that reflects the nurse's proficiency [19].

## Conclusion

This study showed that EHR–generated APCS compared to existing KPCSs can provide useful information on patients’ severity and needs of nursing care to estimate nursing workload. Future studies are needed to develop and implement a real-world, EHR-based PCS system to capture both direct and indirect nursing care, considering factors affecting clinical nursing activities such as bed size, grade of nursing management fee, ratio of severely inpatients, skill mix, and proficiency of the nursing staff.

## Supplementary Information

**Additional file 1.** 

## Data Availability

All the data supporting the study findings are within the manuscript. Additional detailed information and raw data are available from the corresponding author upon reasonable request.

## Abbreviations

ANOVA: Analysis of variance
APCS: Asan Patient Classification System
HER: Electronic health record
Kc: Kappa value
KPCS: Korean Patient Classification System
PCS: Patient classification system
PPS: Pediatric Patient Classification System
