# Supplementary material for: Measuring patient acuity and nursing care needs in South Korea: application of a new patient classification system
Source: BMC Nurs. 2022 Nov 29;21:332. doi: 10.1186/s12912-022-01109-4 (PMC9707110; doi:10.1186/s12912-022-01109-4)
Supplement: Supplementary file 1 — Additional file 1. [file 12912_2022_1109_MOESM1_ESM.docx]

Appendix 1. Description of APCS, KPCS-1, and KPCS-GW

| **APCS** | |  |  | **KPCS-1** | |  |  | **KPCS-GW** | |  |  |
| --- | --- | --- | --- | --- | --- | --- | --- | --- | --- | --- | --- |
| **Domain** | **Nursing activities** | **Appraisal criteria** | **Score** | **Domain** | **Nursing activities** | **Appraisal criteria** | **Score** | **Domain** | **Nursing activities** | **Appraisal criteria** | **Score** |
| 1. Vital sign measurement | Vital sign | 1~3 / 4~6 / 7~9 / ≥ 10 times | 1/2/3/4 | 1. Vital sign measurement | Vital sign | 1~3 / 4~6 / 7~9 / ≥ 10 times | 1/2/3/4 | 1. Vital sign measurement/Monitoring | Vital sign | 1~3 / 4~6 / 7~9 / ≥ 10 times | 1/2/3/4 |
|  | Femoral/Dorsalis pedis artery pulse | One point if Femoral/dorsalis pedis artery pulse checked nursing record is more than four times | 1 |  | Femoral/Dorsalis pedis artery pulse | One point if Femoral/dorsalis pedis artery pulse check is more than four times | 1 |  |  |  |  |
|  | Positional blood pressure |  |  |  |  |  |  |  |  |  |  |
|  | Fetal heart rate | The number of times recorded in the nursing prescription  1~3 /4~6 /7~9 / ≥4 times | 1/2/3/4 |  |  |  |  |  |  |  |  |
| 2. Monitoring | Intake/Output | Intake/Output check  1~2 / 3 / ≥4 times | 1/2/3 | 2. Monitoring | Intake/Output | Intake/Output check  1~2 / 3 / ≥4 times | 1/2/3 |  | Intake/Output | Intake/Output check  1~2 /3 / ≥4 times | 1/2/3 |
|  | Circulation, Motor, Sensory | One point if the nursing course record time is at least four times | 1 |  | Circulation, Motor, Sensory | One point if the nursing course record time is at least four times | 1 |  |  |  |  |
|  | Consciousness, Orientation, Pupil reflex | Two points if there are at least four pupil reflex, consciousness, orientation recorded in the nursing record | 2 |  | Consciousness, Orientation, Pupil reflex | Two points if the pupil reflex, consciousness, and orientation are all recorded | 2 |  | Consciousness, Orientation, Pupil reflex | Two points if the pupil reflex, consciousness, and orientation are all recorded | 2 |
|  | continuous Electrocardiogram monitoring | If there are more than three nursing records or ECG records, three points | 3 |  | continuous Electrocardiogram monitoring | Three points if the ECG reading is recorded at least once per shift | 3 |  | continuous Electrocardiogram monitoring | Three points if the ECG reading is recorded at least once per shift | 3 |
|  | Oxygen saturation | Depending on the number of nursing prescriptions  3~5 / ≥6 times | 1/2 |  | Oxygen saturation | Depending on the number of nursing prescriptions  3~5 / ≥6 times | 1/2 |  | Oxygen saturation | Depending on the number of nursing prescriptions  3~5 / ≥6 times | 1/2 |
|  | Pain assessment | One point if the number of pain records is at least three times | 1 |  | Pain assessment | One point if the number of pain records is at least three times  (Use pain assessment tools) | 1 |  |  |  |  |
|  | NIHSS stroke assessment | Two points if the nursing record used by a stroke assessment tool is at least three times | 2 |  |  |  |  |  |  |  |  |
|  | Central venous pressure | One point if the number of nursing prescriptions is more than three | 1 |  |  |  |  |  |  |  |  |
|  | Intracranial pressure | One point if the nursing prescription is entered | 1 |  |  |  |  |  |  |  |  |
|  | Arterial line monitoring | One point if the nursing prescription is entered | 1 |  |  |  |  |  |  |  |  |
|  | In-bed body weight scale | One point if the nursing prescription is entered | 1 |  |  |  |  |  |  |  |  |
|  | Skin graft site assessment | One point if the nursing prescription is entered | 1 |  |  |  |  |  |  |  |  |
|  | Continuous fetal monitoring before delivery | Two points if there are at least three records in the nursing course | 2 |  |  |  |  |  |  |  |  |
| 3. Respiratory  therapy | Oxygen administration | Two points if nursing prescription is entered | 2 | 3. Respiratory  therapy | Oxygen administration |  | 2 | 2. Respiratory  therapy | Oxygen administration |  | 2 |
|  | Deep breathing training using Incentive spirometry | One points if nursing prescription is entered | 1 |  | Deep breathing training using Incentive spirometry |  | 1 |  |  |  |  |
|  | Chest physiotherapy | Measured by the number of nursing prescriptions  3~5 / ≥6 times | 1/2 |  | Chest physiotherapy | Record after using percussion and vibrator  3~5 / ≥6 times | 1/2 |  | Chest physiotherapy | Record after using percussion and vibrator  3~5 / ≥6 times | 1/2 |
|  | Endotracheal suctioning | Measured by the number of nursing prescriptions  3~5 / ≥6 times | 2/3 |  | Endotracheal suctioning | Number of suction nursing through T-cannula, E-tube  3~5 / ≥6 times | 2/3 |  | Endotracheal suctioning | Number of suction nursing through T-cannula, E-tube  3~5 / ≥6 times | 2/3 |
|  | Tracheostomy care | Two points if there is a nursing prescription or nursing record | 2 |  | Tracheostomy care | T-cannula exchange or dressing | 2 |  | Tracheostomy care | T-cannula exchange or dressing | 2 |
|  | Mechanical ventilator care | Two points if there is a nursing prescription | 2 |  |  |  |  |  |  |  |  |
| 4. Hygiene | Bed bath | Four points if there is a nursing prescription | 4 | 4. Hygiene | Bed bath | Four points if performed by the nurse herself | 4 | 3. Hygiene | Bed bath | Four points if performed by the nurse herself | 4 |
|  | Bed hair wash | Two points if there is a nursing prescription | 2 |  | Bed hair wash | Two points if performed by the nurse herself | 2 |  |  |  |  |
|  | Oral care | One points if there is a  nursing prescription | 1 |  | Oral care |  | 1 |  | Oral care |  | 1 |
|  | Perineal care | One points if there is a  nursing prescription | 1 |  | Perineal care |  | 1 |  | Perineal care |  | 1 |
|  | Exchange of linen and patient clothes | Measured by the number of nursing prescriptions  1~2 / ≥3 times | 1/ 2 |  | Exchange of linen and patient clothes | The number of times the nurse performed it herself  1~2 / ≥3 times | 1/2 |  | Exchange of linen and patient clothes | The number of times the nurse performed it herself  1~2 / ≥3 times | 1/2 |
|  | Bathing a newborn | Four points if there is a nursing prescription | 4 |  |  |  |  |  |  |  |  |
| 5. Feeding | Tube feeding | Measured by the number of nursing prescriptions  1~3 / ≥4 times | 2/4 | 5. Feeding | Tube feeding | 1~3 / ≥4 times | 2/4 | 4. Feeding | Tube feeding | 1~3 / ≥4 times | 2/4 |
|  | Spoon feeding | One point if the nurse has a spoon feeding record | 1 |  |  |  |  |  |  |  |  |
|  | Infant/Neonate bottle feed | Measured by the number of nursing prescriptions | 2 |  |  |  |  |  |  |  |  |
| 6. Excretion | Diaper change | Two points if the number of nursing prescriptions is more than three times | 2 | 6. Excretion | Diaper change | Two points if the nurse directly performed it more than three times | 2 | 5. Excretion | Diaper change | Two points if the nurse directly performed it more than three times | 2 |
|  | Portable toilet/urinal toilet application | Two points if the number of nursing prescriptions is more than three times | 2 |  | Portable toilet/urinal toilet application | Two points if the nurse directly performed it more than three times | 2 |  |  |  |  |
|  | intermittent catheterization | One point if you have a nursing prescription | 1 |  | Intermittent catheterization |  | 1 |  |  |  |  |
|  | enema | One point if you have a nursing prescription | 1 |  | Enema |  | 1 |  |  |  |  |
|  | residual urine measurement | One point if you have a nursing prescription | 1 |  |  |  |  |  |  |  |  |
| 7. Ambulation/activity | change of position | Measured by the number of nursing prescriptions  3~5 / ≥6 times | 2/3 | 7. Ambulation | change of position | The number of times the nurse performed it herself  3~5 / ≥6 times | 2/3 | 6. Ambulation | change of position | The number of times the nurse performed it herself  3~5 / ≥6 times | 2/3 |
|  | patient transfer | Extracted from the transfer application screen when moving from bed to wheelchair or mobile bed  2~3 / ≥4 times | 1/2 |  | patient transfer | Extracted from the transfer application screen when moving from bed to wheelchair or mobile bed  2~3 / ≥4 times | 1/2 |  | patient transfer | Extracted from the transfer application screen when moving from bed to wheelchair or mobile bed  2~3 / ≥4 times | 1/2 |
|  | Assisting a patient to a sitting position and ambulation | Two points if you have a nursing prescription | 2 |  | Assisting a patient to a sitting position and ambulation | Two points if performed by the nurse herself | 2 |  | Assisting a patient to a sitting position and ambulation | Two points if performed by the nurse herself | 2 |
|  | Passive Range of Motion exercises | One point if the number of nursing prescriptions is more than twice | 1 |  |  |  |  |  |  |  |  |
| 8. Examination | Blood glucose test | Scores based on the number of records on the blood sugar record  1~4 / ≥5 times | 1/2 | 8. Examination | Blood glucose test | 1~4 / ≥5 times | 1/2 | 7. Examination | Blood glucose test | 1~4 / ≥5 times | 1/2 |
|  | Additional blood tests (performed by the nurse) | Two points when an examination is received by an additional doctor's prescription other than the regular prescription | 2 |  | Additional blood tests (performed by the nurse) | Two points when an examination is received by an additional doctor's prescription other than the regular prescription | 2 |  | Additional blood tests (performed by the nurse) | Two points when an examination is received by an additional doctor's prescription other than the regular prescription | 2 |
|  | Collect a 24-hour urine test | Two points if the test is received | 1 |  |  |  |  |  |  |  |  |
|  | continuous blood test | Two points if the test is received | 2 |  |  |  |  |  |  |  |  |
|  | nutritional screening tool | One point if you have a doctor's prescription | 1 |  |  |  |  |  |  |  |  |
| 9. Medication/  transfusion | Changing intravenous solution | The number of times the medication was recorded and prescribed by the doctor  1~2 /3~4 / ≥5 times | 1/3/4 | 9. Medication | Changing intravenous solution | 1~2 /3~4 / ≥5 times | 1/3/4 | 8. Medication/  transfusion | Changing intravenous solution | 1~2 /3~4 / ≥5 times | 1/3/4 |
|  | Multi-lumen combined Intravenous administration | One point if you have a nursing prescription | 1 |  | Multi-lumen combined Intravenous administration |  | 1 |  | Multi-lumen combined Intravenous administration |  | 1 |
|  | intravenous medication administration | The number of times it was recorded on the medication record  3~5 /6~9 / ≥10 times | 1/2/3 |  | Intravenous medication administration | The sum of the number of doses per drug administered to patients  3~5 /6~9 / ≥10 times | 1/2/3 |  | intravenous medication administration | The sum of the number of doses per drug administered to patients  3~5 /6~9 / ≥10 times | 1/2/3 |
|  | blood transfusion | Two points per transfusion unit | 2 |  | Blood transfusion | Blood check, vital sign check, patient monitoring. Platelets, Cryo are once per 6 units | 2 |  | blood transfusion | Blood check, vital sign check, patient monitoring. Platelets, Cryo are once per 6 units | 2 |
|  | blood transfusion- Platelet transfusions, Cryoprecipitate | One point per Cryoprecipitate/ Platelet transfusions 3unit | 1 |  |  |  |  |  |  |  |  |
|  | Dosing methods other than intravenous injections | Time of administration of administration records excluding injections  3~5 / 6~10 / ≥11 times | 1/2/3 |  | Dosing methods other than intravenous injections | Number of visits to patients for medication  3~5 / 6~10 / ≥11 times | 1/2/3 |  | Dosing methods other than intravenous injections | Number of visits to patients for medication  3~5 / 6~10 / ≥11 times | 1/2/3 |
| 10. Tube/catheter insertion and management | Peripheral venous catheter insertion | One point if you have a nursing prescription | 1 | 10. Treatment | Peripheral venous catheter insertion | One points if performed by the nurse herself | 1 | 9. Treatment | Peripheral venous catheter insertion | One points if performed by the nurse herself | 1 |
|  | Nasogastric tube insertion and management | One point if you have a nursing prescription | 1 |  | Nasogastric tube insertion and management |  | 2 |  | Nasogastric tube |  | 2 |
|  | Rectal tube insertion and management | Two point if you have a nursing prescription | 2 |  | Rectal tube insertion and management |  | 2 |  | Rectal tube |  | 2 |
|  | Foley catheter insertion | Two point if you have a nursing prescription | 2 |  | Foley catheter insertion |  | 2 |  | Foley catheter |  | 2 |
|  | Central venous catheter insertion | Two points if there is a nursing record or nursing prescription | 2 |  | Central venous catheter insertion |  | 2 |  | Central venous catheter insertion |  | 2 |
|  | Chest tube insertion and management | One points if there is a  nursing prescription | 1 |  | Chest tube insertion and management |  | 3 |  | Chest tube |  | 3 |
|  | Various tube management | Two points if the sum of the number of nursing prescriptions is more than three | 2 |  | Various tube management | These include Penrose drain, JP drain, PCD, Gastrostomy tube, PTBD, H-vac, etc | 3 |  |  |  |  |
|  | Sengstaken-Blakemore/Miller-Abbott tube | Two point if you have a nursing prescription | 2 |  |  |  |  |  |  |  |  |
|  | Arterial line Set up | One points if there is a  nursing prescription | 1 |  |  |  |  |  |  |  |  |
|  | irrigation | One points if there is a  nursing prescription | 1 |  |  |  |  |  |  |  |  |
|  | Chest bottle change | One points if there is a  nursing prescription | 1 |  |  |  |  |  |  |  |  |
| 11. Wound management | Dressing | One points if there is a  nursing prescription | 1 |  |  |  |  |  |  |  |  |
|  | Stoma care | Two points if there is a  nursing prescription | 2 |  |  |  |  |  |  |  |  |
|  | Leech therapy | Two points if you have a nursing record | 2 |  |  |  |  |  |  |  |  |
|  | Heat lamp care | Two points if there is a  nursing prescription | 2 |  |  |  |  |  |  |  |  |
| 12. Treatment | Puncture preparation and nursing | Two points if there is a nursing record or nursing prescription | 2 |  | Puncture preparation and nursing | Preparation and assistance for lumbar puncture, thoracetesis, etc | 2 |  |  |  |  |
|  | Nursing care before surgery and procedures | Three points if there is a record of nursing before surgery or examination | 3 |  | Nursing care before surgery and procedures | Confirmation of consent for surgery or procedure, skin preparation, medication and training description, etc. | 3 |  | Nursing care before surgery and procedures | Confirmation of consent for surgery or procedure, skin preparation, medication and training description, etc. | 3 |
|  | Treatments that take more than 15 minutes | Two points if there is a nursing prescription | 2 |  | Treatments that take more than 15 minutes | Two points if performed by the nurse herself | 2 |  | Treatments that take more than 15 minutes | Two points if performed by the nurse herself | 2 |
|  | Cardiopulmonary Cerebral Resuscitation | 6 points if there is a nursing record for CPR implementation | 6 |  | Cardiopulmonary Cerebral Resuscitation | CPR implementation | 6 |  | Cardiopulmonary Cerebral Resuscitation | CPR implementation | 6 |
|  | Isolation requiring the use of a gown or mask | Records of quarantine targets registered with Alert | 2 |  | Isolation requiring the use of a gown or mask |  | 2 |  |  |  |  |
|  | restraint application care | Two points if there is a  nursing prescription | 2 |  | Restraint application care | Monitoring the circulation of the restraint area | 2 |  |  |  |  |
|  | cold and hot therapy | Two points if there is a nursing prescription | 2 |  | Cold and hot therapy |  | 2 |  |  |  |  |
|  | Pediatric sedation | Two points if you have a sleep sedative prescription | 2 |  |  |  |  |  |  |  |  |
|  | Skeletal traction: extremities | Two points if there is a  nursing prescription | 2 |  |  |  |  |  |  |  |  |
|  | Rupture of (fetal) membrane test | One point if the sum of nursing/doctor's prescription is more than two times | 1 |  |  |  |  |  |  |  |  |
|  | Nursing of painless labor through epidural analgesia | One points if there is a  nursing prescription | 1 |  |  |  |  |  |  |  |  |
|  | Luminaria insertion | Measured according to the number of nursing prescriptions  1 times/2 times/≥3 times | 1  2  3 |  |  |  |  |  |  |  |  |
|  | Bakri (intrauterine balloon) insertion | One points if there is a  nursing prescription | 1 |  |  |  |  |  |  |  |  |
|  | Knot remove | One points if there is a  nursing prescription | 1 |  |  |  |  |  |  |  |  |
|  | Vaginal gauze packing | One points if there is a  nursing prescription | 1 |  |  |  |  |  |  |  |  |
|  | Newborn nursing care | One points if there is a  nursing prescription | 1 |  |  |  |  |  |  |  |  |
| 13. Admission and discharge | Nursing admission | Five points for inpatients | 2 | 11. Special treatment | Nursing admission | Inpatient nursing information survey sheet, physical measurement, education, etc | 5 | 10. Admission management | Nursing admission | Inpatient nursing information survey sheet, physical measurement, education, etc | 5 |
|  | Transfer/discharge nursing | Three points for transfer/discharge nursing | 3 |  | Transfer/discharge nursing | Transfer nursing: Transfer between wards, discharge nursing: discharge education and administrative affairs, etc | 3 |  |  |  |  |
|  | End-of-life care | Five points if you are registered for the management of End-of-life | 5 |  | End-of-life care |  | 5 |  |  |  |  |
| 14. Others (Education, emotional support, and other things) | Education (15 minutes or more) | Two points if there is a nursing prescription | 2 | 12. Education/ emotional support | Education (15 minutes or more) | Patient education by disease | 2 | 11. Education/ emotional support | Education(15 minutes or more) | Patient education by disease | 2 |
|  | Emotional support for the patient and family (more than 15 minutes) | Two points if there is a nursing prescription | 2 |  | Emotional support for the patient and family (more than 15 minutes) |  | 2 |  | Emotional support for the patient and family (more than 15 minutes) |  | 2 |
|  | Communication Disorder Nursing | Three points if there is a nursing prescription | 3 |  | Communication Disorder Nursing | When communicating with a patient with visual/aural/verbal loss requires more time and effort than an average patient | 3 |  | Communication Disorder Nursing |  | 3 |
|  | Dialysis Nursing | Two points if there is a nursing prescription | 2 |  |  |  |  |  |  |  |  |
|  | Breast Nursing | Two points if there is a nursing prescription | 2 |  |  |  |  |  |  |  |  |
|  | uterine massage | Two points if there is a nursing prescription | 2 |  |  |  |  |  |  |  |  |
|  | Postural drainage | One points if there is a  nursing prescription | 1 |  |  |  |  |  |  |  |  |
|  | Infant phototherapy/infant circumcision | One points if there is a  nursing prescription | 1 |  |  |  |  |  |  |  |  |
|  | Pediatric febrile seizure | One points if there is a  nursing prescription | 1 |  |  |  |  |  |  |  |  |
|  | Breastfeeding management | One points if there is a  nursing prescription | 1 |  |  |  |  |  |  |  |  |
|  | Seizure disorder nursing care | One points if there is a  nursing record | 1 |  |  |  |  |  |  |  |  |
|  | Labor and delivery nursing care | Three points if there is a  nursing prescription | 3 |  |  |  |  |  |  |  |  |
|  | Newborn nursing care | Two points if you have a newborn registration number | 2 |  |  |  |  |  |  |  |  |

APCS: Asan Patient Classification System; KPCS-1, Korean Patient Classification System-1; KPCS-GW, Korean Patient Classification System-General Ward
